# *MTHFD2* is a potential oncogene for its strong association with poor prognosis and high level of immune infiltrates in urothelial carcinomas of bladder

**DOI:** 10.1186/s12885-022-09606-0

**Published:** 2022-05-17

**Authors:** Lin Zhu, Xianhui Liu, Weiyu Zhang, Hao Hu, Qi Wang, Kexin Xu

**Affiliations:** 1 Department of Urology, Peking University People’s Hospital, 11 Xizhimen South Street, Xicheng District, Beijing, 100044 China; 2 Peking University Applied Lithotripsy Institute, Peking University People’s Hospital, Beijing, 100034 China

**Keywords:** Urothelial carcinomas of bladder, *MTHFD2*, The Cancer Genome Atlas website, Biomarker, Immune infiltrates, Prognosis

## Abstract

**Background:**

The bifunctional methylenetetrahydrofolate dehydrogenase (NADP+ dependent) 2, methenyltetrahydrofolate cyclohydrolase (*MTHFD2*) has been reported to play an oncogenic role in various types of cancers. However, the function of *MTHFD2* in urothelial carcinomas of bladder (UCB) and its association with tumor immune infiltration remains unknown. We aim to examine the suitability of *MTHFD2* to be a novel biomarker of bladder cancer and whether *MTHFD2* is linked to immune infiltration.

**Methods:**

RNA sequencing data and clinical information (bladder cancer samples: normal samples = 414: 19) were downloaded from The Cancer Genome Atlas official website. Western blot analysis was performed to detect MTHFD2 expression in human bladder cancer (BLCA) cells and normal urothelial cell line SV-HUC-1. Associations between *MTHFD2* expression and clinicopathological features were analyzed using Mann Whitney U test or Kruskal-Wallis H test. The “survival” and “survminer” packages were utilized to plot Kaplan-Meier survival curves. Moreover, the gene set enrichment analysis (GSEA) was conducted using a clusterProfiler package. The correlation of *MTHFD2* expression with immune infiltration level was estimated using the single sample GSEA (ssGSEA) algorithm. Furthermore, associations between *MTHFD2* and immune checkpoint genes were evaluated using the correlation analysis*.*

**Results:**

Transcriptome analysis manifested that *MTHFD2* was highly expressed in UCB tissues than normal bladder tissues, which was further confirmed by western blot analysis in human BLCA cells and SV-HUC-1 cells. Moreover, *MTHFD2* high expression was significantly associated with the advanced disease progression. Also, the high expression of *MTHFD2* was correlated with poor prognosis, and *MTHFD2* was considered as an independent prognostic factor for disease specific survival. Furthermore, a number of cancer-related pathways were enriched in *MTHFD2* high group, including NF-κB activation, JAK/STAT, and cancer immunotherapy by PD1 blockade. Several immune checkpoint molecules were also strongly associated with *MTHFD2* expression, including *PDCD1*, *CD274*, *CTLA4*, *CD276*, *LAG3*, *HAVCR2*, and *TIGIT*.

**Conclusions:**

*MTHFD2* expression was remarkably elevated in UCB, suggesting that MTHFD2 could be a promising biomarker for BLCA as well as novel target for anti-cancer immunotherapy since its close association with immune infiltration.

**Supplementary Information:**

The online version contains supplementary material available at 10.1186/s12885-022-09606-0.

## Background

Bladder cancer (BLCA) is the fourth most prevalent cancer expected to be diagnosed in men in the United States, with estimated 61,700 new cases and 12,120 deaths in 2022 [1]. The incidence rate in males is 3 to 5 times higher than that in females [2]. It is obvious that patients suffer a lot from this kind of disease which needs lifelong surveillance (with cystoscopy, bladder biopsy, and urine cytology) and invasive treatment. At the same time, remarkable morbidity and mortality of BLCA contribute to tremendous economic burdens on families and health care systems, causing serious social problems [3]. The major histology type of BLCA is urothelial carcinomas, where non-muscle-invasive BLCA (NMIBC) accounts for approximately 75% and the remaining is muscle-invasive BLCA (MIBC) [4]. The treatment regimens and efficacy of BLCA may be influenced by multiple factors, such as the disease stage, grade, associated risk factors, and other clinical characteristics. The current standard treatment for NMIBC is transurethral resection (TUR), followed by intravesical mitomycin C or bacillus Calmette-Guerin (BCG) if necessary [5]. For MIBC, the front-line treatment is neoadjuvant chemotherapy combined with radical cystectomy [6]. Besides, MIBC is more aggressive and serious because 5-year survival rate of MIBC patients with localized disease is 60% and that of patients with metastases is less than 10% [7].

Urothelial carcinomas of the bladder (UCB) are histologically classified as high-grade bladder urothelial carcinoma (HGBC) and low-grade bladder urothelial carcinoma (LGBC). HGBC is more likely to have a high risk of recurrence, progression, and metastasis than LGBC. To our knowledge, both histological grade and tumor-node-metastasis (TNM) stage are closely related to prognosis [5, 8]. However, there is no significant molecular marker able to predict prognosis or direct the treatment of UCB. Therefore, effective and reliable molecular markers are in high demand.

Previous literatures have proposed several potential prognostic factors for UCB, including urinary biomarkers, tissue biomarkers, blood biomarkers. Serial measurements of UroVysion FISH in urine were reported to have strong relationship with both cancer recurrence and progression. AUA/SUO expert opinion pointed that a clinician may use UroVysion FISH to assess response to intravesical BCG [9, 10]. For bladder tissue biomarkers, nuclear accumulation of *p53* was found prognostic in MIBC, as well as inactivating mutation of *Rb* combined with other cell cycle regulatory proteins [11, 12]. *Survivin* and *Bcl-2* family were also reported to be associated with bladder cancer recurrence and mortality among patients who performed radical cystectomy [13]. Meanwhile, *FGFR3, GATA3, FOXA1, ERBB2, ERCC2, CDKN2A, STAG2,CDH1,CDH3,*estrogen receptor-β etc., were reported to characterize patients with MIBC into distinct molecular subtypes, which could potentially predict prognosis [14, 15]. Insulin growth factor binding proteins (IGFBPs), transforming growth factor–β1(TGF-β1) and VEGF, as UCB blood biomarkers, were illustrated to be associated with adverse survival outcomes [16]. However, these biomarkers have not yet been applied to clinical use as they are still unable to guide clinical decision-making in UCB. Thus, we intend to uncover more potential molecular candidates for UCB. The bifunctional methylenetetrahydrofolate dehydrogenase (NADP+ dependent) 2, methenyltetrahydrofolate cyclohydrolase (*MTHFD2*) is a mitochondrial enzyme that assumes a pivotal role in one-carbon folate metabolic. It was recently reported as a potential target for anticancer therapy. *MTHFD2* has been observed to exert oncogenic effects in a variety of cancers. The non-metabolic functions of *MTHFD2* in cancers have aroused considerable research interests. The high level of *MTHFD2* has been shown to be connected with the poor prognosis of solid and hematological malignancies. For colorectal cancer cell lines, *MTHFD2* could facilitate cell proliferation, migration, and cycle entry, and suppress cell apoptosis [17]. In breast cancer, *MTHFD2* was reported to activate the AKT signaling pathway to promote tumorigenesis [18]. Meanwhile, *MTHFD2* could accelerate tumorigenesis and metastasis via the AKT/glycogen synthase kinase-3β (GSK-3β)/β-catenin signaling pathway in lung adenocarcinoma [19] and via the STAT3 signaling pathway in ovarian cancer [20]. Recently, MTHFD2 was found to affect bladder cancer cell growth by activating CDK2 [21]. All these observations indicated that MTHFD2 is tightly associated with the tumorigenesis of various cancer types.

However, the functional importance of *MTHFD2* is still poorly investigated in BLCA. Intuitively, we suspect that *MTHFD2* might play a role in BLCA progression as well as its prognosis. In this study, we provided lines of evidences to elucidate the correlation between *MTHFD2* and prognosis of patients with UCB. For immune infiltrates, *MTHFD2* expression was positively correlated with T helper 2 (Th2) cells, Th1 cells, macrophages, activated dendritic cells (aDCs), and T gamma delta (Tgd) immune cells. We conclude that MTHFD2 could be a promising biomarker for BLCA as well as a novel target for anti-cancer immunotherapy due to its close association with immune infiltration.

## Materials and methods

### Data acquisition

RNA sequencing (RNA-seq) data and clinical information were downloaded from BLCA project level 3 HTSeq-Fragments Per Kilobase per Million (FPKM) in The Cancer Genome Atlas (TCGA, https://portal.gdc.cancer.gov/). FPKM data were converted to transcripts per million (TPM) data, and corresponding values were transformed using log2. Four hundred fourteen patients (tumor samples: normal samples = 414:19) with both RNA-seq data and clinical information were enrolled in this study. RNA-seq datasets from various bladder cancer cell lines such as UMUC10 and BFTC905, were downloaded from the Cancer Cell Line Encyclopedia (CCLE).

### Cell lines and cell culture

Human BLCA cell lines (BIU-87, 5637, EJ, T24, and TCCSUP) and normal urothelial cell line SV-HUC-1 were purchased from National Infrastructure of Cell Line Resource (Beijing, China). BIU-87, 5637, and EJ cell lines were maintained in Roswell Park Memorial Institute 1640 medium (Gibco, Carlsbad, California, USA) encompassing 10% fetal bovine serum (Gibco) and penicillin/streptomycin (Gibco).

T24 and TCCSUP cell lines were cultured in complete Dulbecco’s Modified Eagle Media (Gibco), and SV-HUC-1 cell line was incubated in complete Ham’s F12K (Kaighn’s) modified medium (Gibco). All cells were cultured in a humidified CO_2_ condition at 37 °C.

### Protein extraction and western blot analysis

Proteins were extracted with Radio-Immunoprecipitation assay buffer (Shanghai Biotechwell, Shanghai, China), and protease and phosphatase inhibitors were added immediately before use. The protein concentration in the supernatant was measured using a bicinchoninic acid assay kit (Shanghai Biotechwell). Western blot analysis was performed following standard procedures. The primary antibodies employed in this study were MTHFD2 antibody (1:1000, mouse, Abcam) and β-actin antibody (1:2000, mouse, Shanghai Biotechwell). Secondary antibodies were horseradish peroxidase-coupled goat anti-mouse antibodies (12,000, Jackson ImmunoResearch Laboratories Inc., West Grove, PA, USA).

### Tumor immune estimation

The tumor immune estimation resource, version 2 (TIMER2.0) website (http://timer.cistrome.org/) can robustly estimate the connection of immune infiltrates with other factors, such as gene expression, mutation status, somatic copy-number variants, and clinical outcome. Meanwhile, users could explore cancer-related associations based on TCGA [22]. We predicted the expression difference of *MTHFD2* between tumor and normal tissue in different tumors from “Gene_DE” module of TIMER2.0.

Gene Set Variation Analysis (GSVA) was an unsupervised gene set enrichment method that can be applied for detecting subtle pathway activity changes within highly heterogeneous data sets [23]. The correlation of *MTHFD2* expression with immune infiltration level (24 types of immune cells) was estimated using the single sample gene set enrichment analysis (ssGSEA) algorithm by an R GSVA package. The analysis was conducted with the use of the different markers of various immune cells from the literature published in Cell [24]. The R ggplot2 package was adopted to assess the association between *MTHFD2* expression and immune checkpoint molecule expression, including programmed cell death 1 (*PDCD1*), *CD274*, cytotoxic T lymphocyte-associated antigen 4 (*CTLA4*), *CD276*, colony-stimulating factor 1 receptor (*CSF1R*), indoleamine 2,3 dioxygenase-1 (*IDO1*), lymphocyte activation gene 3 (LAG3), hepatitis A virus cellular receptor 2 (*HAVCR2*), and T cell immunoglobulin and immunoreceptor tyrosine-based inhibitory motif domain (*TIGIT*)*.*

### siRNA transfection

siRNA sequences used to knock down MTHFD2 level were chemically synthesized by Sangon Biotech (Shanghai) Co., Ltd. The siRNA sequences were as follows (MTHFD2-SI-17: sense-CGAAUGUGUUUGGAUCAGUAUTT, antisense-AUACUGAUCCAAACACAUUCGTT; MTHFD2-SI-36: sense-CGAGAAGUGCUGAAGUCUAAATT, antisense-UUUAGACUUCAGCACUUCUCGTT; NC: sense-UUCUCCGAACGUGUCACGUTT, antisense-ACGUGACACGUUCGGAGAATT).

One day before transfection, plate cells in 24-well plate with 500 μl of growth medium without antibiotics. At the time of transfection, cells will be 50–70% confluent. For each well to be transfected, RNAi duplex-Lipofectamine RNAiMAX complexes were prepared as follows: a. Dilute 6 pmol RNAi duplex in 50 μl Opti-MEM Medium without serum. Mix gently. b. Mix Lipofectamine RNAiMAX gently before use, then dilute 1 μl in 50 μl Opti-MEM Medium. Mix gently. c. Combine the diluted RNAi duplex with the diluted Lipofectamine™ RNAiMAX. Mix gently and incubate for 10–20 min at room temperature. Add the RNAi duplex-Lipofectamine RNAiMAX complexes to each well containing cells. This gives a final volume of 600 μl and a final RNA concentration of 10 nM. Mix gently by rocking the plate back and forth. Change medium after harvesting 4–6 h and incubate the cells 24–48 h at 37 °C in a CO2 incubator until we carry out further experiments.

### Cell proliferation and cell circle analysis

Cell proliferation was measured using the Cell Counting Kit-8(CCK8), which was purchased from Dojindo Molecular Technologies. We performed the experiment following manufacturer-recommended procedures. Cells were seeded at a density of 1 × 10^4/mL in 96-well plates. We record the absorbance at 450 nm at 0 h,24 h,48 h,72 h, 96 h using a microplate reader. Cell circle analysis was assessed by flow cytometry according to standard protocols. Briefly, cells were washed twice in PBS, stained with propidium iodide (PI) solution for 30 min at 37 °C, analyzed by flow cytometry using FlowJo 10.

### Immunohistochemical staining

Tumor and normal tissues of three BLCA patients were obtained from Peking University People’s Hospital with ethics committee approval. We performed immunohistochemical staining steps followed as each instruction of products. The tissues were dewaxed in xylene, rehydrated in graded ethanol and incubated with EDTA (pH = 9.0, Maxim Biotech) buffer for antigen retrieval. Endogenous peroxidase was blocked by 6% hydrogen peroxide for 10 min. After washing with PBS three times, sections were incubated with primary antibody (MTHFD2, 1:100, mouse, Abcam) overnight at 4 °C. Then sections were further incubated with a secondary antibody EnVision™+/HRP labelled anti-rabbit antibody (Dako) for half an hour. DAB method was used for color development.

Two pathologists were blinded to the clinical information of specimens, they classified staining intensity into 4 grades: 0 (no staining of cancer cells), 1 (weak staining), 2 (moderate staining), and 3 (strong staining). The percentage of positive cells was divided into 5 grades: 0–4 (0, < 25%, 26–50%, 51–75, > 76%). The IHC score was determined as the staining intensity multiplying the percentage of positive cells.

### Statistical analysis

R(v.3.6.3) was applied for all data processing and statistical analysis. RNA-seq analysis of differential expression in UCB tissues (*n* = 414) and normal bladder tissues (*n* = 19) was performed with DEseq2. Genes with an absolute log2 fold change (log2FC) > 0.7 and adjusted *p*-value (*p*.adjust) <  0.01 were considered as differentially expressed genes. Then we ranked 56,493 genes from UCB samples in TCGA by their relative *MTHFD2* expression in the top 50th percentile and the bottom 50th percentile for GSEA using a clusterProfiler package. We perform GSEA analysis of essential pathways in UMUC10 and BFTC905 cell lines in the same way. The C2.cp.v7.2.symbols.gmt was utilized for GSEA with the random combinatorial count set as 1000. Gene set collections were acquired from MsigDB. Moreover, false discovery rate (FDR) <  0.25 with *p*.adjust < 0.05 was considered significant.

The association between *MTHFD2* expression and clinicopathological features was analyzed by the Mann Whitney U test or the Kruskal-Wallis H test using a ggplot2 package. The probabilistic receiver-operating characteristic (pROC) Package was applied to plot ROC curve. For Kaplan-Meier survival curves, the survival and survminer packages were utilized to conduct the survival analysis based on *MTHFD2* expression along with some clinical features. We determined the cut-off value of *MTHFD2* expression by its median. *p* <  0.05 was considered to be significant (ns, *p* ≥ 0.05; *, *p* <  0.05; **, *p* <  0.01; ***, *p* <  0.001).

## Results

### Clinical characteristics of patients with UCB

The clinical information of 414 patients with UCB in TCGA is presented in Table 1. There were 234 (56.5%) patients less than or equal to 70 years old at the time of diagnosis and 180 (43.5%) patients over 70 years old, with 109 (26.3%) females and 305 (73.7%) males. As for race information, 330 (83.1%) patients were white, 44 (11.1%) were Asian, and 23 (5.8%) were black or African American. In terms of body mass index, 153 (42.0%) patients were less than or equal to 25, and 211 (58.0%) patients were over 25. As for subtype, there exited 134 (32.8%) patients with non-papillary UCB and 275 (67.2%) patients with papillary UCB. Among these patients, 292 (72.8%) patients were smokers, and the other 109 (27.2%) patients were not.

**Table 1:** Clinical characteristics of patients with urothelial carcinomas of the bladder in TCGA

Characteristic	Total	Percentage (%)
n	414	
Age		
≤ 70	234	56.5
> 70	180	43.5
Gender		
Female	109	26.3
Male	305	73.7
Race		
White	330	83.1
Asian	44	11.1
Black or African American	23	5.8
BMI		
≤ 25	153	42.0
> 25	211	58.0
Tumor subtype		
Papillary	275	67.2
Non-papillary	134	32.8
Smoker		
No	109	27.2
Yes	292	72.8
T stage		
T1	5	1.3
T2	119	31.3
T3	196	51.6
T4	60	15.8
N stage		
N0	239	64.6
N1, N2, N3	131	35.4
M stage		
M0	202	94.8
M1	11	5.2
Histologic grade		
Low grade	21	5.1
High grade	390	94.9
Pathologic stage		
Stage I	4	1.0
Stage II	130	31.6
Stage III	142	34.5
Stage IV	136	33.0

Overall, 5 (1.3%) patients were at T stage I, 119 (31.3%) patients at stage II, 196 (51.6%) patients at stage III, and 60 (15.8%) patients at stage IV. Additionally, 239 (64.6%) patients were diagnosed with clear N stage, and 131 (35.4%) patients had lymph node metastases. Distant metastasis occurred in 11 (5.2%) patients, and the other 202 (94.8%) patients showed no distant metastasis. There were 21 (5.1%) patients diagnosed with low grade and 390 (94.9%) patients with high grade. The pathologic stage of 412 patients was available, among which 4 (1.0%) patients were at stage I, 130 (31.6%) patients at stage II, 142 (34.5%) patients at stage III, and 136 (33.0%) patients at stage IV. The median follow-up of the 414 patients alive at the time of the last contact was 21.2 months (ranged from 0 to 169 months).

### *MTHFD2* was upregulated in various cancer types including UCB

**Fig. 1:**
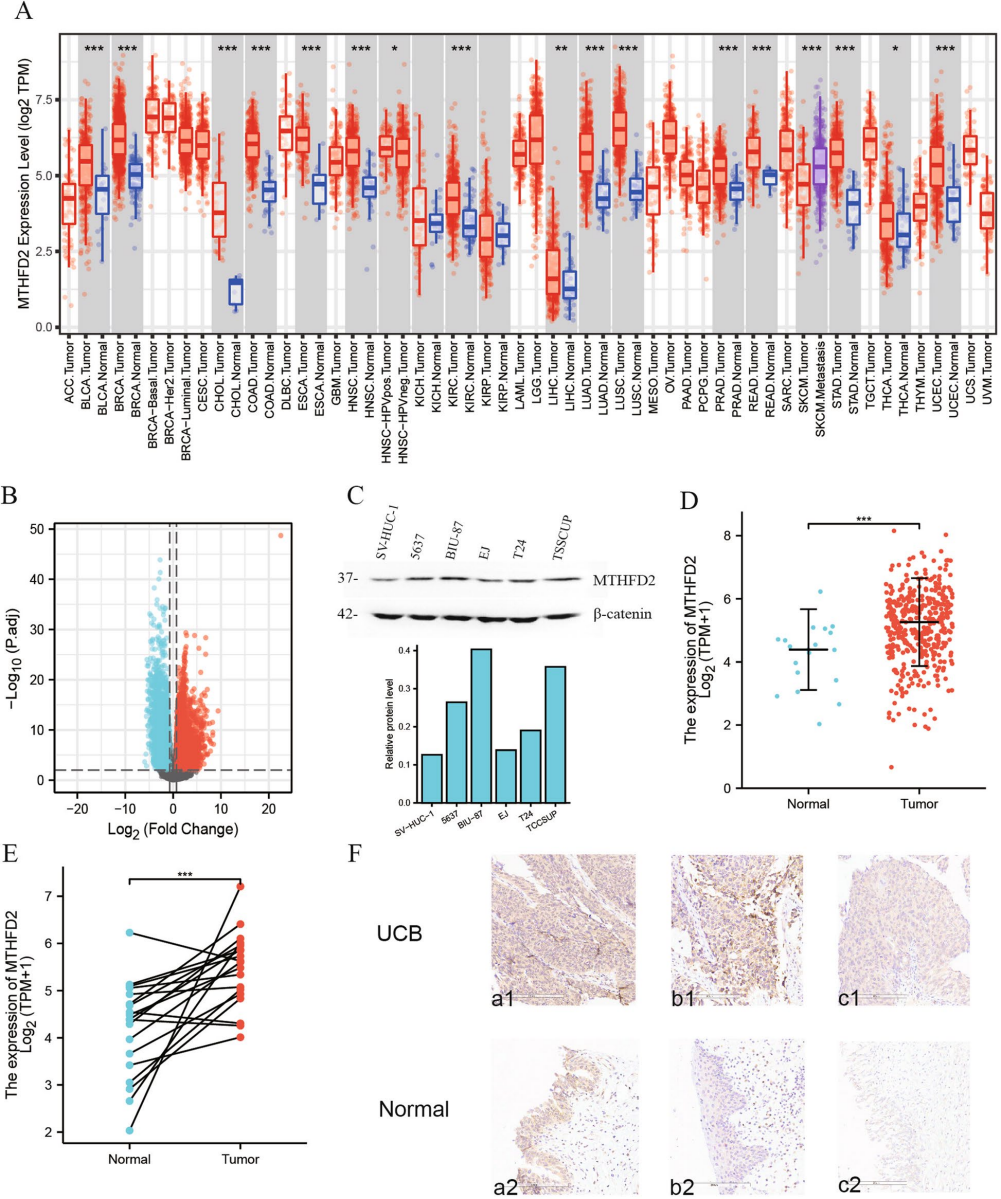
MTHFD2 expression in various cancers notably in bladder cancer (ns, *p* ≥ 0.05; *, *p* <  0.05; **, *p* <  0.01; ***, *p* <  0.001). **A** Differential expression of MTHFD2 in various types of cancer analyzed by TIMER2.0. **B** The volcano plot of RNA-Seq analysis of differential expression in UCB tissues and normal bladder tissues. **C** Higher expression of MTHFD2 in 5637, BIU-87, EJ, T24, and TCCSUP cells than in SV-HUC-1 cells validated by western blot analysis. **D** Differential expression of MTHFD2 in UCB tissue samples and normal bladder tissue samples. **E** Differential expression of MTHFD2 in UCB tissue samples and their paired normal bladder tissue samples. **F** Immunohistochemistry (IHC) for the tumor and normal tissues of three BLCA patients. MTHFD2 was used for staining

As reflected by TIMER analysis, *MTHFD2* expression was considerably augmented in multiple types of cancers, like BLCA, breast cancer, cholangiocarcinoma, colon adenocarcinoma, and kidney renal clear cell carcinoma (Fig. 1A). Through RNA-seq analysis of differential expression, upregulated genes were filtered by log2FC > 0.7 and FDR <  0.01in UCB, and notably *MTHFD2* was considered to be statistically significant (log2FC = 0.789 and FDR = 0.002) (Fig. 1B, Table S1). Furthermore, the results of western blot analysis manifested higher expression of MTHFD2 in human BLCA cells (BIU-87, 5637, EJ, T24, and TCCSUP) than SV-HUC-1 cells (Fig. 1C). We also performed quantification gel analysis on the WB results. We obtained that the densities of BLCA cell bands was significantly higher than that of the SV-HUC-1 (control) cells band (Fig. 1C). By comparing 414 UCB samples and 19 normal bladder tissue samples from RNA-seq data, the unpaired tissue contrast displayed that *MTHFD2* expression was elevated in UCB samples (*p* = 3.8e-4) (Fig. 1D). The same results were seen in the comparison between 19 UCB samples and their paired normal bladder tissue samples (*p* value = 1.6e-4) (Fig. 1E). In order to make a robust conclusion, we performed immunohistochemistry (IHC) for the tumor and normal tissues of three BLCA patients. MTHFD2 was used for staining. It is clearly seen that the tumor tissues have higher MTHFD2 expressions than normal tissues (Fig. 1F), confirming that the *MTHFD2* gene expression is elevated in BLCA (Table.S1_F).

### The association of *MTHFD2* expression with clinical features of patient

**Fig. 2:**
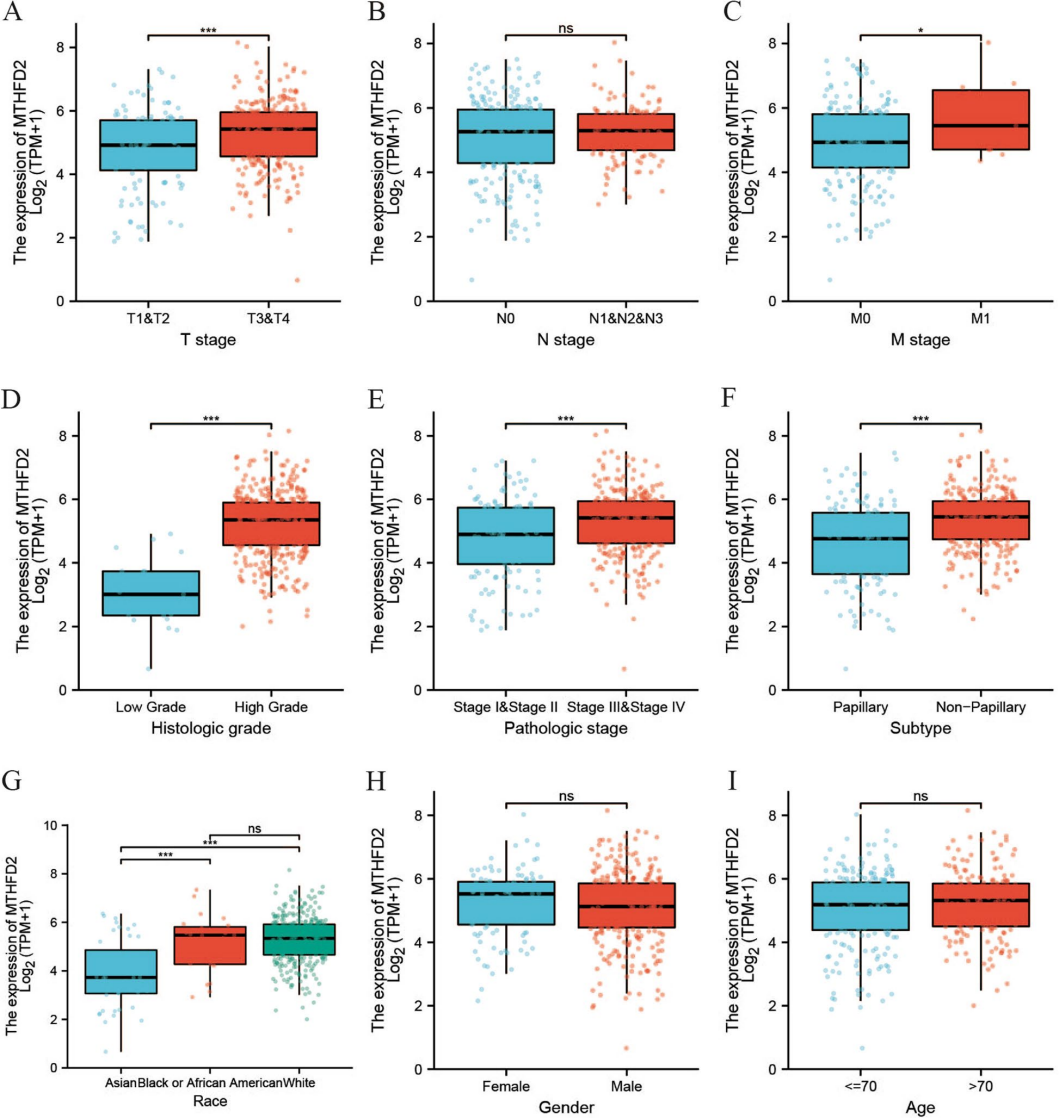
Associations of MTHFD2 expression with clinicopathological characteristics of patients (ns, *p* ≥ 0.05; *, *p* <  0.05; ***, *p* <  0.001). **A** T stage. **B** N stage. **C** M stage. **D** Histologic grade. **E** Pathologic stage. **F** Tumor subtype (papillary& non-papillary). **G** Race. **H** Gender. **I** Age

Moreover, *MTHFD2* expression was strongly correlated with T stage (T1&2 vs. T3&4, *p* <  0.001; Fig. 2A), M stage (M0 vs. M1, *p* = 0.03; Fig. 2C), histologic grade (low grad vs. high grade, *p* <  0.001; Fig. 2D), pathologic stage (stage I-II vs. stage III-IV, *p* <  0.001; Fig. 2E), tumor subtype (papillary vs. non-papillary, *p* <  0.001; Fig. 2F), and human race (*p* <  0.001; Fig. 2G). Meanwhile, no significant difference was observed between groups stratified by N stage (N0 vs. N1&N2&N3, *p* = 0.41; Fig. 2B), gender (female vs. male, *p* = 0.19; Fig. 2H), and age (≤ 70 years old vs. > 70 years old, *p* = 0.35; Fig. 2I).

### Survival analysis and cox regression analysis

**Fig. 3:**
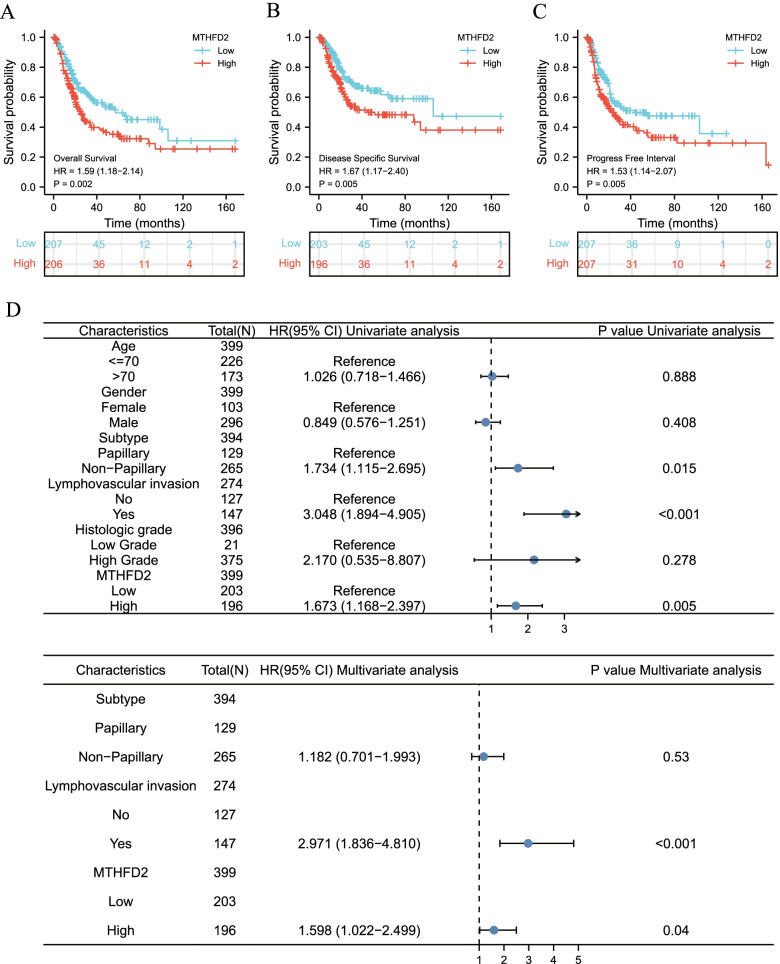
Survival analysis and forest plot of multivariate Cox regression analysis. The influence of MTHFD2 expression on survival was analyzed: **A** Overall survival. **B** Disease-specific survival. **C** Progress-free interval. **D** Forest plot of univariate and multivariate Cox regression analysis

Kaplan-Meier survival analysis documented that the high expression of *MTHFD2* evidently diminished the overall survival (OS, *p* = 0.002, Fig. 3A), disease-specific survival (*p* = 0.005, Fig. 3B), and progress-free interval (*p* = 0.005, Fig. 3C) of patients with UCB. Table 2 and Fig. 3D depicted the results of univariate and multivariate Cox regression analyses for theDSS of patients with UCB. From the univariate Cox regression model, subtype [papillary versus non-papillary, the hazard ratio (HR) = 1. 734 (1.115–2.695), *p* = 0.015)], lymphovascular invasion [HR =3.048 (1. 894–4.905), *p* <  0.001], and MTHFD2 expression [HR = 1.673 (1.168–2.397), p = 0.005] were associated with the DSS of patients with UCB (Fig. 3D). Missing values were removed from further analysis, and remaining samples were included. Subsequent to adjustment of multivariate Cox regression. Lymphovascular invasion [HR = 2.971 (1.836–4.810), p <  0.001], and MTHFD2 expression [HR = 1.598 (1.022–2.499), *p* = 0.04] were considered to be independent prognostic factors for the DSS of patients with UCB (Fig. 3D).

**Table 2:** Univariate and multivariate Cox regression analysis on disease-specific survival

Characteristics	Total(N)	Univariate analysis		Multivariate analysis	
		Hazard ratio (95% CI)	*P* value	Hazard ratio (95% CI)	*P* value
Age	399				
< =70	226	Reference			
> 70	173	1.026 (0.718–1.466)	0.888		
Gender	399				
Female	103	Reference			
Male	296	0.849 (0.576–1.251)	0.408		
Subtype	394				
Papillary	129	Reference			
Non-Papillary	265	1.734 (1.115–2.695)	**0.015**	1.182 (0.701–1.993)	0.530
Lymphovascular invasion	274				
No	127	Reference			
Yes	147	3.048 (1.894–4.905)	**< 0.001**	2.971 (1.836–4.810)	**< 0.001**
Histologic grade	396				
Low Grade	21	Reference			
High Grade	375	2.170 (0.535–8.807)	0.278		
MTHFD2	399				
Low	203	Reference			
High	196	1.673 (1.168–2.397)	**0.005**	1.598 (1.022–2.499)	**0.040**

*CI* Confidence interval

### Illustration of the high expression level of of *MTHFD2* by GSEA

**Fig. 4:**
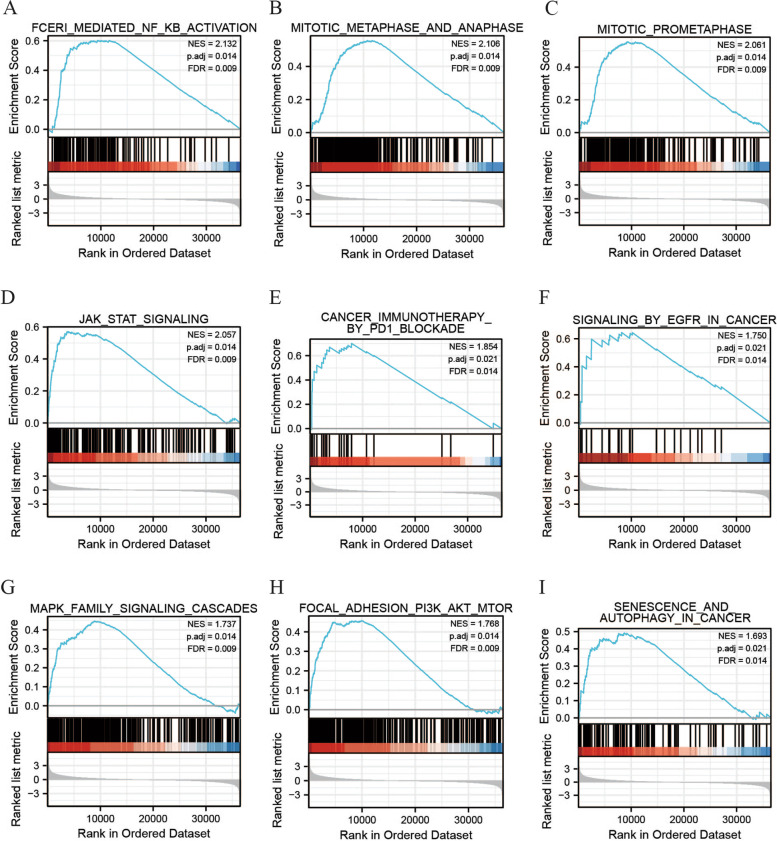
Enrichment plots from gene set enrichment analysis. Gene sets enriched in MTHFD2 high expression phenotype were analyzed: **A** NF-κB activation. **B** Mitotic metaphases and anaphase. **C** Mitotic prometaphases. **D** JAK/STAT. **E** Cancer immunotherapy by PD1 blockade. **F** Signaling by EGFR in cancer. **G** MAPK family signaling cascades. **H** PI3K/AKT/mTOR. **I** Senescence and autophagy in cancer pathways

Totally 508 gene sets were found to be substantially enriched in *MTHFD2* high expression phenotype from different pathway databases (Table S2). Partial gene sets of interest were visualized. We focused on those gene sets which were associated with cancer growth, progression, and metastasis, such as nuclear factor κB (NF-κB) activation, mitotic phases, Janus kinase/signal transducer and activator of transcription (JAK/STAT), cancer immunotherapy by programmed death 1 (PD1) blockade, signaling pathway by epidermal growth factor receptor (EGFR) in cancer, mitogen-activated protein kinase (MAPK) family signaling cascades, phosphatidylinositol 3-kinase (PI3K)/AKT/mammalian target of rapamycin (mTOR), senescence, and autophagy in cancer pathways (Fig. 4).

**Fig. 5:**
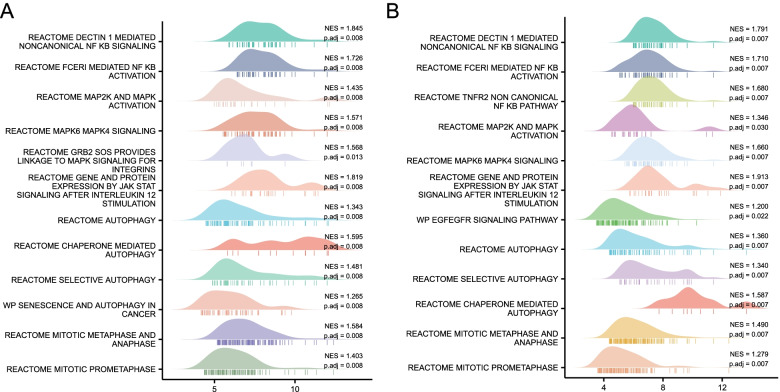
Expression of essential pathways in UMUC10 (**A**) and BFTC905 cell lines (**B**). *P* value < 0.05 indicates the expression of genes within this pathway is significantly enriched in this dataset

For further validation of our assumption, we downloaded two sets of RNA-Seq expression data from CCLE (Cancer Cell Line Encyclopedia). These two data sets came from the UMUC10 and BFTC905 cell lines (two BLCA cell lines), respectively. The essential pathways like the MAP, STAT, NF-KB, cell cycle and autophagy pathways were examined (Fig. 5A and B). They also exhibited significantly high expression levels and thus were activated in BLCA cells.

### MTHFD2 expression was correlated with immune infiltrates

**Fig. 6:**
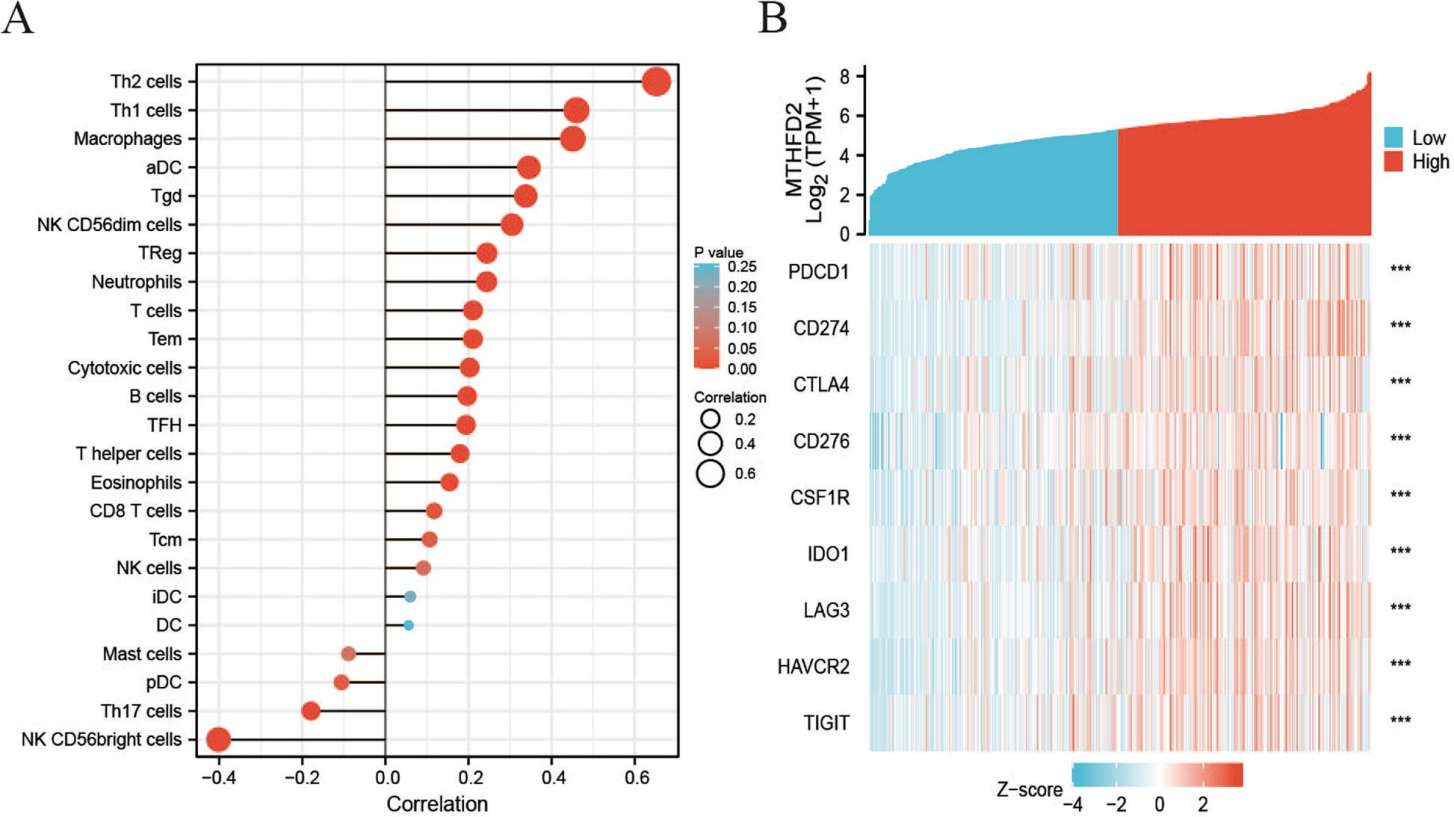
Immune infiltrate assessment. **A** Correlation of MTHFD2 expression with immune cell infiltration. **B** Correlation of MTHFD2 expression with immune checkpoint molecule expression

Lollipop diagrams indicated that *MTHFD2* expression shared strong positive correlation with Th2 cells (Spearman correlation; rho = 0.647, *p* < 0.001), Th1 cells (Spearman correlation; rho = 0.457, *p* < 0.001), macrophages (Spearman correlation; rho = 0.450, *p* < 0.001), aDC (Spearman correlation; rho = 0.341, *p* < 0.001), and Tgd (Spearman correlation; rho = 0.341, *p* < 0.001) immune cells. However, CD56bright natural killer cells (Spearman correlation; rho = − 0.394, *p* < 0.001) exhibited close negative correlation with *MTHFD2* expression (Table 3, Fig. 6A). Furthermore, *MTHFD2* expression was also strongly associated with immune checkpoint molecules reported in previous studies [25, 26], including *PDCD1* (Spearman correlation; rho = 0.357, *p* < 0.001), *CD274* (Spearman correlation; rho = 0.532, *p* < 0.001), *CTLA4* (Spearman correlation; rho = 0.373, *p* < 0.001), *CD276* (Spearman correlation; rho = 0.406, *p* < 0.001), *CSF1R* (Spearman correlation; rho = 0.387, *p* < 0.001), *IDO1* (Spearman correlation; rho = 0.372, *p* < 0.001), lymphocyte activation gene 3 (*LAG3*) (Spearman correlation; rho = 0.471, *p* < 0.001), *HAVCR2* (Spearman correlation; rho = 0.464, *p* < 0.001), and *TIGIT* (Spearman correlation; rho = 0.353, *p* < 0.001) (Fig. 6B).

**Table 3:** Correlation analysis between MTHFD2 expression and immune checkpoint molecule expression

Gene_name	Immune cells	Rho (Spearman)	*p* value (Spearman)
MTHFD2	Th2 cells	0.647	< 0.001
MTHFD2	Th1 cells	0.457	< 0.001
MTHFD2	Macrophages	0.450	< 0.001
MTHFD2	NK CD56bright cells	-0.394	< 0.001
MTHFD2	aDC	0.341	< 0.001
MTHFD2	Tgd	0.341	< 0.001
MTHFD2	NK CD56dim cells	0.298	< 0.001
MTHFD2	Neutrophils	0.242	< 0.001
MTHFD2	TReg	0.239	< 0.001
MTHFD2	Tem	0.216	< 0.001
MTHFD2	T cells	0.213	< 0.001
MTHFD2	Cytotoxic cells	0.198	< 0.001
MTHFD2	B cells	0.195	< 0.001
MTHFD2	T helper cells	0.187	< 0.001
MTHFD2	TFH	0.187	< 0.001
MTHFD2	Th17 cells	−0.169	< 0.001
MTHFD2	Eosinophils	0.159	0.001
MTHFD2	CD8 T cells	0.113	0.022
MTHFD2	pDC	−0.108	0.028
MTHFD2	Tcm	0.108	0.027
MTHFD2	NK cells	0.087	0.078
MTHFD2	Mast cells	−0.082	0.095
MTHFD2	iDC	0.056	0.254
MTHFD2	DC	0.054	0.273

### CCK8 and FACS experiments verified the oncogenic role of MTHFD2

**Fig. 7:**
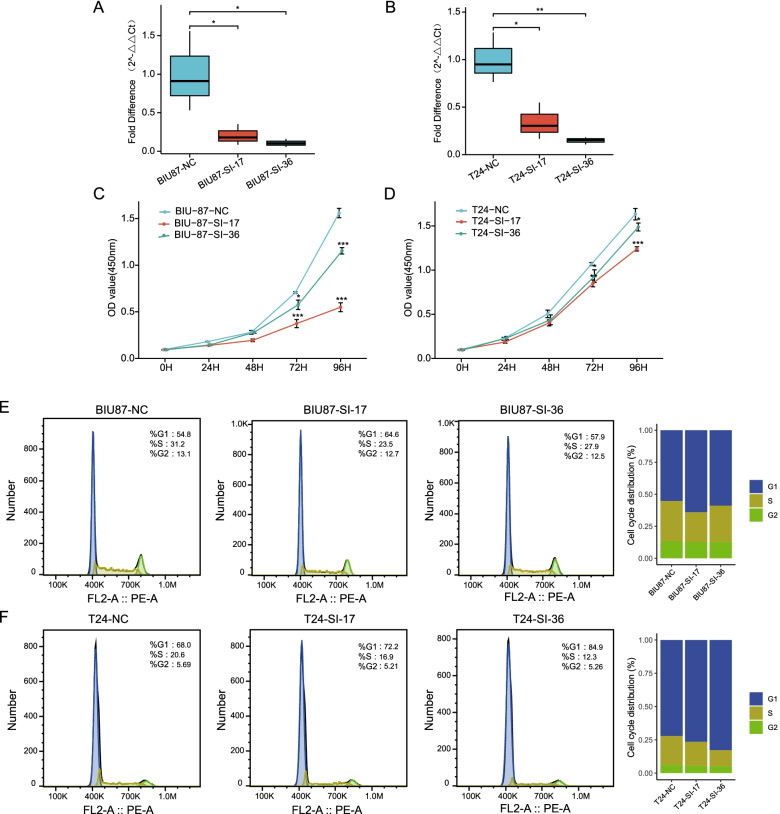
Experimental verification of the oncogenic role of MTHFD2 gene. **A**, **B** Knock-down of MTHFD2 by two different siRNAs in two BLCA cell lines. Significance was calculated by. One-way ANOVA *, *P* < 0.05; **, *P* < 0.01. **C**, **D** The OD value at different time points. Significance was calculated by Two-way repeated measures ANOVA. *, P < 0.05; **, P < 0.01; ***, *P* < 0.001. **E**, **F** FACS (Fluorescence-activated Cell Sorting) of control and si-MTHFD2 cells. Higher fraction in G1 phase represents a less actively dividing state of cells which means less likely to promote oncogenesis

To further experimentally verify the oncogenic role of MTHFD2 gene, we knocked down MTHFD2 with two siRNAs in two BLCA cell lines, respectively (Fig. 7A and B). The OD value detection revealed that the control cells progressed significantly faster than the two si-MTHFD2 cell lines (Fig. 7C and D), suggesting that MTHFD2 might contribute to BLCA cell growth/proliferation. Next, we performed FACS (Fluorescence-activated Cell Sorting) of control and si-MTHFD2 cells. We consider that a higher fraction of cells in G1 (gap1) phase represents a less actively dividing state of cells which means less likelihood to promote oncogenesis, while a higher fraction in S (synthesis) phase indicates more dividing cells. Not surprisingly, we observed that control cells had discernibly lower fractions of cells (~ 5% difference) in G1 phase (Fig. 7E and F). This suggests that MTHFD2 might have promoted the transition from G1 to S phase to regulate cell cycle and consequently contribute to the oncogenesis of bladder cancer.

## Discussion

Given the fact that the currently known biomarkers of UCB are still unable to be applied to clinical practice or provide any guidance for the subsequent treatment, it is necessary to discover a novel and reliable biomarker. Early diagnosis of UCB is the key to improve the prognosis of patients. Plenty of researches have reported some prognostic biomarkers for BLCA, including protein biomarkers, genomic biomarkers, epigenetic biomarkers, and transcriptomic biomarkers, such as fibroblast growth factor receptor 3 (*FGFR3*), Telomerase reverse transcriptase (*TERT*) mutation, One cut domain family member 2 (*ONECUT2*), polymerase (RNA) III (DNA directed) polypeptide G (*POLR3G*), Cyclin A1 (*CCNA1*), B cell lymphoma 2 (*BCL2*), Eomesodermin (*EOMES*), Vimentin (*VIM*), and high-temperature requirement A serine peptidase 1 (*HtrA1*) [27, 28] . Our study results indicated that *MTHFD2* was a potential prognostic biomarker of UCB patients, which can be a new choice to be applied in the clinic.

*MTHFD2* is a bifunctional enzyme existing in mitochondrion with nicotinamide adenine dinucleotide-specific dehydrogenase activity [29]. *MTHFD2* takes crucial part in mitochondrial folate-mediated one-carbon pathway, and meanwhile, it is consistently overexpressed in embryos and a wide range of tumors [30].

Previous study reported that Carolacton (a natural product of MTHFD2 inhibitor tool compound) was able to restain the growth of different human tumor cell lines [31]. Then, an isozyme-selective *MTHFD2* inhibitor, DS18561882, was discovered to repress the proliferation of breast cancer cell lines and the tumor growth in a mouse xenograft model with breast carcinoma upon oral administration [32]. The regulatory factors and mechanisms of elevated *MTHFD2* expression in various cancer types need to be clarified, since *MTHFD2* is a promising novel target for anti-cancer therapy [33].

The functions of *MTHFD2* in UCB have not been previously reported clearly, so we wonder if *MTHFD2* also plays a crucial part in BLCA. Our study elaborated that *MTHFD2* was highly expressed in various types of tumors, including UCB. The expression of *MTHFD2* in UCB tissues was evidently higher than that in normal bladder tissues. We could get the same conclusion in the comparison of both unpaired and paired bladder tissue samples. The result of western blot showed that *MTHFD2* expression was higher in UCB cell lines than in the SV-HUC-1 cell line. IHC analysis further confirmed higher expression in BLCA tissue than paired normal bladder tissue. Through these results, we knew that during UCB tumorigenesis, *MTHFD2* expression is a cumulative alteration.

Our study uncovered that *MTHFD2* upregulation is noticeably associated with high T stage, high M stage, high histologic grade, and high pathologic stage. Those clinical features all predicted the poor clinical outcome. Furthermore, *MTHFD2* expression is potently enhanced in non-papillary UCB. It is obvious that compared to papillary subtype, non-papillary UCB is more invasive and that most of them could cause shortened survival due to metastatic spread [34]. Then, we conducted Kaplan Meier survival analysis, univariate analysis, and multivariate analysis on the DSS. The result manifested that lymphovascular invasion, and high *MTHFD2* expression were considered to be correlated with the poor prognosis, and that *MTHFD2* was regarded as an independent prognostic factor for the DSS in patients with UCB.

GSEA was further implemented to decipher the mechanism of *MTHFD2* functioning in UCB, the result of which suggested that numerous signaling pathways were enriched in *MTHFD2* high expression group, including NF-κB activation, mitotic phases, JAK/STAT, cancer immunotherapy by PD1 blockade, signaling pathway by EGFR in cancer, MAPK family signaling cascades, PI3K/AKT/mTOR, and senescence and autophagy in cancer pathways. The essential pathways like the MAP, STAT, NF-KB, and autophagy pathways were also exhibited significantly high expression levels and were activated in BLCA cells such as UMUC10 and BFTC905. Some of them were already reported in other carcinomas by prior works [17–20, 30]. Our follow-up studies interrogated the molecular mechanisms of *MTHFD2* in UCB to find a potential anti-cancer target.

Immunotherapy is a vital part of clinical treatment for UCB. Intravesical BCG has been regarded as the most effective immunotherapy for NMIBC due to its approval by U.S. Food and Drug Administration (FDA) in 1990, which is proved to reduce recurrence and prolong survival [35, 36]. As for metastatic UCB, immune checkpoint inhibitors have recently manifested to have good activity and significant efficacy. The first target was CTLA4 that could interact with CD80/86 to restrict T-cell activation [37]. Ipilimumab and tremelimumab, the monoclonal antibodies targeting CTLA4, were developed. Some scientists found that drugs directly against either PD1 or PD-L1 had lower adverse effects than CTLA4 inhibitors for their direct action in tumor microenvironment [38]. The interaction of the PD1 signaling pathway, such as the PD1 receptor and its 2 ligands, activated a cascade of events and decreased cytokine secretion, T-cell activation, and targeted cell lysis [26]. PD1/PD-L1 antibodies, therefore, improved antitumor immunity. PD-L1 inhibitors, Atezolizumab and Durvalumab, were both approved by FDA for the treatment of patients with inoperable or metastatic UCB progressing on platinum-based treatment with positive PD-L1 [39]. A PD1 inhibitor, Pembrolizumab, has been approved in the first-line treatment of UCB patients ineligible for cisplatin with tumor PD-L1 positive [40]. These immune checkpoint inhibitors were exhibited to have significant efficacy and bring obvious survival benefits to patients with metastatic UCB [39].

Above all, the level of immune infiltration is apparently associated with the UCB prognosis. Meanwhile, immune infiltration can be a prognostic indicator of immunotherapy response. Therefore, it is a crucial part of treatment for UCB to identify valid predictive biomarkers for immune checkpoint inhibitor therapy. We noticed that cancer immunotherapy by PD1 blockade pathway was considerably enriched in *MTHFD2* high expression group by GSEA. Then we wonder if *MTHFD2* expression is correlated with immune infiltrates.

From the lollipop diagrams, we knew that *MTHFD2* expression was positively correlated with Th2 cells, Th1 cells, macrophages, aDC, and Tgd immune cells. It was reported that the efficient response to BCG in UCB required Th1-type immunity, and that Th2-promoting factors could trigger Th1-type immunity contributing to the treatment of BCG [41]. Secondly, tumor-associated macrophages had two functional states including M1 (anti-tumor) and M2 (tumor-promoting). The increased ratio of M1/M0 might correlate to the improved prognosis of UCB [42]. In prior researches, the high level of CD8+ T cells was indicated to trigger the improved prognosis and the augmented possibility of response to immunotherapy, which was the basic mechanism of immune checkpoint inhibitors [43–45]. Of note, CD4+ T cells and dendritic cells were also illustrated to be supportive in this process [46]. Taken together, *MTHFD2* expression was significantly associated with the level of those infiltrating immune cells which were indicators of disease prognosis and assumed a central role in immunotherapy for UCB. Moreover, we found that several immune checkpoint molecules exhibited close association with *MTHFD2* expression, including *PDCD1*, *CD274*, *CTLA4*, *CD276*, *CSF1R*, *IDO1*, *LAG3*, *HAVCR2*, and *TIGIT*. In summary, *MTHFD2* might be a new target of anti-tumor immunity, which contributes to the orchestration of immune cell infiltrates and the expression of immune checkpoint molecules. This potential mechanism could demonstrate the reason why improved prognosis was observed in patients with poor *MTHFD2* expression. The *MTHFD2* inhibitors may provide an effective way for the treatment of UCB.

As the results of CCK8 and FACS experiments shows, MTHFD2 might contribute to BLCA cell growth/proliferation via regulating cell cycle. Our following study will continue to figure out if *MTHFD2* could drive tumor cell proliferation, migration, and cycle progression and inhibit cell apoptosis in UCB, just as in other tumors, and to study the potential direct or indirect mechanism. Furthermore, we will research the anti-tumor work of the *MTHFD2* inhibitors to find a selective target for immunotherapy.

### To our best knowledge, this is the first study demonstrating the prognostic role and immune infiltration level of *MTHFD2* in UCB

We concluded that *MTHFD2* was an independent prognostic factor for the DSS of patients with UCB and could be a potential biomarker of disease prognosis. *MTHFD2* also might be a new target of anti-tumor immunity for its strong association with immune infiltration. There are some limitations in this study. Firstly, the clinical data downloaded from TCGA was limited and incomplete. Our study findings need to be validated in larger and multi-center cohort studies. Secondly, the prognostic ability of *MTHFD2* in UCB was lack of validation from adequate clinical samples. Otherwise, the anti-tumor mechanisms of *MTHFD2* and the potential targets for immunotherapy in UCB need to be elucidated via molecular experiments.

## Conclusions

Above all, *MTHFD2* expression was elevated in UCB, which could be a potential biomarker of disease prognosis. *MTHFD2* also might be a promising novel target for anti-cancer immunotherapy for its strong correlation with immune infiltration.

## Supplementary Information

**Additional file 1.**

**Additional file 2.**

**Additional file 3.**

**Additional file 4.**

## Data Availability

The datasets generated during and/or analyzed during the current study are available in the [TCGA] repository, [https://portal.gdc.cancer.gov/], Cancer Cell Line Encyclopedia (CCLE) (broadinstitute.org).

## Abbreviations

AUA: American Urological Association
aDCs: Activated dendritic cells
BCG: Bacillus Calmette-Guerin
BCL2: B cell lymphoma 2
BLCA: Bladder cancer
CCNA1: Cyclin A1
CTLA4: Cytotoxic T-lymphocyte protein 4
CSF1R: Macrophage colony-stimulating factor 1 receptor
EGFR: Epidermal growth factor receptor
EOMES: Eomesodermin
FDR: False discovery rate
FGFR3: Fibroblast growth factor receptor 3
FPKM: Fregments Per Kilobase per Million
GSEA: Gene set enrichment analysis
ssGSEA: Single sample gene set enrichment analysis
GSK-3β: Glycogen synthase kinase-3
GSVA: Gene Set Variation Analysis
HAVCR2: Hepatitis A virus cellular receptor 2
HGBC: High-grade bladder urothelial carcinoma
HR: Hazard ratio
HtrA1: High-temperature requirement A serine peptidase 1
IDO1: Indoleamine 2,3-dioxygenase 1
JAK/STAT: Janus kinase/signal transducer and activator of transcription
LAG3: Lymphocyte activation gene 3
LGBC: Low-grade bladder urothelial carcinoma
log2FC: Log2 fold change
MAPK: Mitogen-activated protein kinase
*MTHFD2*: Methylenetetrahydrofolate dehydrogenase (NADP+ dependent) 2, methenyltetrahydrofolate cyclohydrolase
MIBC: Muscle-invasive bladder cancer
mTOR: Mammalian target of rapamycin
NF-κB: Nuclear factor κB
NMIBC: Non-muscle-invasive bladder cancers
ONECUT2: One cut domain family member 2
OS: Overall survival
PD1: Programmed death 1
PDCD1: Programmed cell death protein 1
PI3K: Phosphatidylinositol 3-kinase
POLR3G: Polymerase (RNA) III (DNA directed) polypeptide G
pROC: Probabilistic receiver-operating characteristic
RNA-seq: RNA sequencing
SUO: Society of Urologic Oncology
TCGA: The Cancer Genome Atlas
TERT: Telomerase reverse transcriptase
Tgd: T gamma delta
Th2: T helper 2
TIMER2.0: Tumor immune estimation resource, version 2
TIGIT: T cell immunoglobulin and immunoreceptor tyrosine-based inhibitory motif domain
TNM: Tumor-node-metastasis
TPM: Transcripts per million
TUR: Transurethral resection
UCB: Urothelial carcinomas of bladder
VIM: Vimentin
