# Supplementary material for: MTHFD2 is a potential oncogene for its strong association with poor prognosis and high level of immune infiltrates in urothelial carcinomas of bladder
Source: BMC Cancer. 2022 May 17;22:556. doi: 10.1186/s12885-022-09606-0 (PMC9112551; doi:10.1186/s12885-022-09606-0)
Supplement: Supplementary file 4 — Additional file 4. [file 12885_2022_9606_MOESM4_ESM.docx]

**Fig.1(C)**


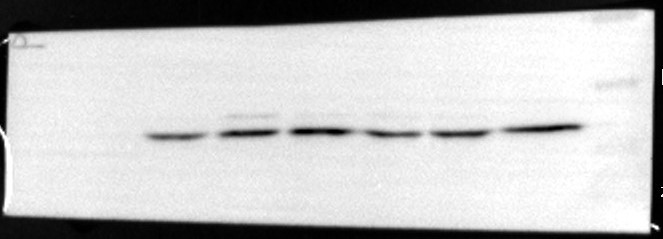


TCCSUP

T24

EJ

BIU-87

5637

SV-HUC-1

25kd

35kd

37kd

40kd

55kd

**MTHFD2**


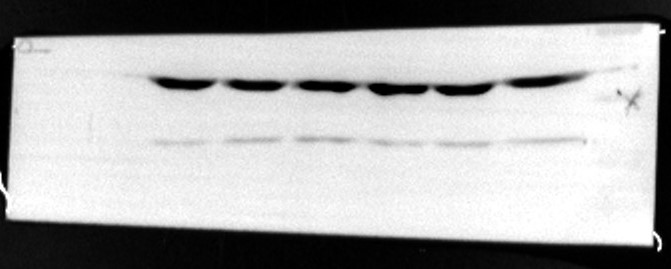


**β-catenin**

EJ

BIU-87

5637

SV-HUC-1

TCCSUP

T24

42kd

25kd

55kd

35kd

40kd

**Fig.1 (C)** Higher expression of MTHFD2 in 5637, BIU-87, EJ, T24, and TCCSUP cells than in SV-HUC-1 cells validated by western blot analysis.
